# Retropharyngeal Hematoma Causing Airway Compromise After Tissue Plasminogen Activator Administration: A Case Report

**DOI:** 10.5811/cpcem.1602

**Published:** 2023-08-09

**Authors:** Christian Provenza, Arun Christian Habermann, Theron Williams, Jeffrey Metter, James Richard Walker

**Affiliations:** * University of Tennessee Health Science Center - Department of Emergency Medicine, Memphis, Tennessee; † University of Tennessee Health Science Center College of Medicine, Memphis, Tennessee; ‡ University of Tennessee Health Science Center – Department of Neurology, Memphis, Tennessee

**Keywords:** Retropharyngeal hematoma, tissue plasminogen activator, stroke, airway, hemorrhage

## Abstract

**Introduction:**

Tissue plasminogen activator (tPA), commonly used for treatment of acute ischemic stroke, is associated with life-threatening bleeding intracranially as well as surrounding the airway.

**Case Report:**

A 78-year-old year old male who presented with stroke symptoms and after tPA administration developed a retropharyngeal hematoma requiring intubation and surgical intervention.

**Conclusion:**

Numerous threats to the patient’s airway can develop after tPA administration. While angioedema is the most common cause, it is important to be prepared for other causes related to hemorrhage.

## INTRODUCTION

The risk of hemorrhage after tissue plasminogen activator (tPA) administration in the treatment of acute ischemic stroke is well known, with intracranial hemorrhage the primary concern.^1^ Tissue plasminogen activator has also been known to cause airway compromise due to angioedema, occurring in 1.3–5.1% of patients.^2^,^3^ While rare, there have also been reported cases of tPA administration leading to hemorrhage causing airway compromise through lingual hematoma and thyroid hemorrhage.^4^–^6^

Head trauma within the prior three months and other major trauma within 14 days as contraindications to tPA administration are Class III, level of evidence C and class IIb, level of evidence C recommendations, respectively.^1^ This is because tPA administration comes with the possibility of worsening or recurrence of traumatic bleeding leading to exsanguination, hemorrhagic shock, or significant hematoma formation due to uncontrolled bleeding. Neck trauma is often associated with clinically insignificant retropharyngeal hematomas, but in rare instances it may be severe enough to cause airway obstruction.^7^,^8^ Retropharyngeal hematomas may also be confused for angioedema in certain clinical settings after tPA administration, given that angioedema is a well-documented phenomenon and thus is high on the differential. We present the case of a hemorrhagic airway obstruction resulting from tPA administration in the setting of minor trauma.

## CASE REPORT

A 78-year-old male with a history of hypertension and hyperlipidemia came to the emergency department (ED) due to right-sided arm weakness. He was initially found on the floor by family after falling out of bed. He could not recall specifically whether he had been weak prior to the fall or if he had fallen and then become weak. Upon arrival to the ED, he was last known normal approximately 94 minutes prior. On exam, the patient had right-sided facial weakness, right upper extremity drift, and right lower extremity drift for a National Institutes of Health Stroke Scale (NIHSS) score of five.

**Image 1:**
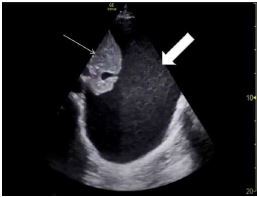
Sagittal slice of the computed tomography of the patient’s cervical spine prior to administration of tissue plasminogen activator. The arrow identifies the level of the patient’s anterior longitudinal ligament rupture and source of his future bleeding.

**Image 2:**
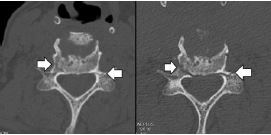
Bilateral sixth cervical vertebra pedicle fractures (arrows) on arrival (left) and post-intubation (right). This image shows the initial subtle fractures that appear similar to vascular channels compared to the clear fractures after intubation.

Computed tomography (CT) of the head showed no acute finding and no intracranial hemorrhage. The CT of the cervical spine, given his fall, showed multilevel degenerative but no acute findings (Images 1 and 2). A CT angiogram showed severe stenosis of the proximal right vertebral artery with occlusion at the V2 segment with retrograde filling at V4 level. His labs on arrival were unremarkable. He was administered tPA approximately three hours after his last known normal.

The patient improved significantly after tPA administration, with resolution of his facial asymmetry and right lower extremity drift, and improvement in his right upper extremity drift for an NIHSS of 1. However, three hours later while holding in the ED, he complained of difficulty breathing. He was tripoding with labored respirations, stridor, and tachycardia, saturating 70% on room air and then 80% on nonrebreather mask. The circumference of his neck was also visibly expanding alarmingly. The initial concern was tPA-induced angioedema, and he was given methylprednisolone, diphenhydramine, and famotidine empirically. However, on examination of the patient’s upper oropharynx, there was no visible evidence of mucosal swelling. He was unable to protect his airway, and given his worsening respiratory status he was emergently intubated by the emergency physician without waiting for a response to medical intervention. During intubation, there was significant narrowing of the patient’s posterior oropharynx and larynx.

**CPC-EM Capsule**

What do we already know about this clinical entity?

*Fibrinolytics can cause hemorrhage. Tissue plasminogen activator (tPA) can cause angioedema and airway compromise.*

What makes this presentation of disease reportable?

*This relatively rare cause of airway compromise after tPA administration has not been previously reported in the literature.*

What is the major learning point?

*Be aware of sources of bleeding other than the brain, other threats to the airway than aniogedema, and the risks after fibrinolytics in all trauma, even minor cases.*

How might this improve emergency medicine practice?

*This case report highlights the potential for post-fibrinolytic complication even after minor trauma.*

**Image 3:**
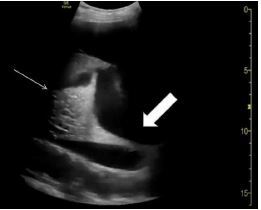
Sagittal slice of magnetic resonance imaging of the patient’s retropharyngeal hematoma, demonstrating total occlusion of the airway at the midline. The endotracheal tube can be visualized above and below the hematoma at this slice. The arrow identifies the ruptured anterior longitudinal ligament and source of the patient’s hematoma. The cervical 5–6 disc space is noticeably wider when compared to cmputed tomography imaging obtained before administration of tissue plasminogen activator (Image 1).

Repeat head CT did not show any bleeding, but given his stiff, swollen neck and deep oropharyngeal swelling, a CT soft tissue of the neck was obtained, which showed a large retropharyngeal hematoma. Image 3 depicts magnetic resonance imaging of the hematoma. Otolaryngology was consulted, and tPA reversal was initiated with cryoprecipitate.

While planning incision and drainage and tracheostomy, the otolaryngologist noticed a cervical spine fracture that was missed on the initial non-contrast CT cervical spine imaging at arrival due to its subtle appearance (Image 2) and the CT soft tissue neck after intubation, as the radiologist had focused on the soft tissue and prevertebral hematoma. The patient was placed in a cervical collar, and radiology reviewed the imaging obtained after intubation, finding a C4 spinous process fracture, widening of the C5–6 anterior disc space suggesting disruption of the anterior longitudinal ligament, C6 right transverse process fracture, and bilateral C6 pedicle fractures. Magnetic resonance imaging (MRI) of the cervical spine confirmed the C5–6 anterior longitudinal ligament rupture, which appeared to be the source of the retropharyngeal hematoma. Neurosurgery was consulted.

The patient then underwent incision and drainage of the retropharyngeal hematoma and tracheostomy, followed immediately by C5–6 open reduction, anterior diskectomy, arthrodesis, and anterior instrumentation. The patient did well postoperatively. There was no further bleeding. An MRI of the brain obtained postoperatively showed new, right-sided cerebellar infarctions.

## DISCUSSION

Major trauma is a relative contraindication to tPA administration.^1^ In this patient who had suffered a minor fall from a height of several feet, cervical fractures and signs of a ligamentous injury were subtle and missed on initial presentation. However, given his significant right-sided weakness and an initially benign interpretation of the CT of the cervical spine, the benefits of tPA administration were determined to outweigh the risks.

Retropharyngeal hematomas are common in trauma patients, especially in the setting of cervical injuries. It has been reported that up to 60% of patients with cervical injury had widening of the prevertebral space, suggestive of retropharyngeal hematoma.^7^ However, airway obstruction due to retropharyngeal hematoma occurs in only 1.2% of patients.^8^ Hyperextension injuries of the cervical vertebrae can cause tearing of the longus colli muscle or anterior longitudinal ligament, which was identified as the likely source of bleeding in this patient.^9^,^10^ Fractures of cervical vertebrae may also damage surrounding soft tissue and tear small branches of vertebral arteries; however, no contrast extravasation was noted on this patient’s CT angiograms for his stroke evaluation.^11^

Tsao et al. performed a systematic review of traumatic retropharyngeal hematoma cases, discovering that 68% of patients with retropharyngeal hematomas had an associated cervical spinal injury, including cervical spinal fracture or dislocation and ligament injury. Their review found that the median age of reported cases was 72 years, that geriatric patients constituted the largest proportion of all patients, and that most (67.6%) were associated with ground-level or low-energy falls. A high index of suspicion for retropharyngeal hematoma should be applied to geriatric patients with trauma, even for those who have sustained only minor trauma.^12^ The elderly patient we describe here with a low-energy fall mechanism and associated cervical spine injury fits all these categories.

The primary threat to life in retropharyngeal hematoma is airway obstruction. Therefore, securing a patent airway is the critical action. In the setting of an acutely obstructed airway, oral intubation, even with video laryngoscope, may be difficult. It is important to have backup equipment including bronchoscope and cricothyroidotomy or tracheostomy equipment on hand. This also applies to airway-threatening angioedema.

As soon as the airway is secure, bleeding should be addressed. The American Heart Association/American Stroke Association (AHA/ASA) recommend tranexamic acid (TXA) or aminocaproic acid, as well as cryoprecipitate, for treatment of intracranial bleeding after tPA administration.^13^ The AHA/ASA guidelines do not make specific recommendations for other sources of bleeding; however, given that the recommendations are specific to the mechanism of tPA, they should be followed for any severe or symptomatic bleeding. Tissue plasminogen activator converts plasminogen to plasmin, which lyses fibrin and fibrinogen, breaking up blood clots. Tranexamic acid and aminocaproic acid directly oppose this mechanism by binding to plasminogen and preventing its conversion to plasmin. The guidelines also recommend cryoprecipitate to replenish fibrinogen levels until its level is greater than 150 milligrams per deciliter to facilitate clotting and hemostasis, as tPA depletes fibrinogen specifically.^13^

## CONCLUSION

It is important to recognize that although angioedema is likely the most common threat to a patient’s airway after tPA administration, clinicians should maintain suspicion for other bleeding threats as well. Careful consideration should be given to the risks of bleeding after tPA administration in the setting of trauma, even when the degree of trauma appears to be minor. Bleeding is a well-known potential complication of tPA and requires emergent reversal by TXA or aminocaproic acid and fibrinogen replacement with cryoprecipitate administration if severe, and swift airway protection is crucial to prevent morbidity and mortality.
